# Directed dynamical influence is more detectable with noise

**DOI:** 10.1038/srep24088

**Published:** 2016-04-12

**Authors:** Jun-Jie Jiang, Zi-Gang Huang, Liang Huang, Huan Liu, Ying-Cheng Lai

**Affiliations:** 1 School of Electrical, Computer and Energy Engineering, Arizona State University, Tempe, AZ 85287, USA; 2 School of Physical Science and Technology, Lanzhou University, Lanzhou 730000, China; 3 School of Computing, Informatics, Decision Systems Engineering, Arizona State University, Tempe, AZ 85287, USA; 4 Department of Physics, Arizona State University, Tempe, Arizona 85287, USA

## Abstract

Successful identification of directed dynamical influence in complex systems is relevant to significant problems of current interest. Traditional methods based on Granger causality and transfer entropy have issues such as difficulty with nonlinearity and large data requirement. Recently a framework based on nonlinear dynamical analysis was proposed to overcome these difficulties. We find, surprisingly, that noise can counterintuitively enhance the detectability of directed dynamical influence. In fact, intentionally injecting a proper amount of asymmetric noise into the available time series has the unexpected benefit of dramatically increasing confidence in ascertaining the directed dynamical influence in the underlying system. This result is established based on both real data and model time series from nonlinear ecosystems. We develop a physical understanding of the beneficial role of noise in enhancing detection of directed dynamical influence.

Two types of behaviors can show similar trends and may thus be highly correlated, but they may not have anything to do with each other. Three hundred years ago already, Bishop Berkeley famously declared that “correlation does not imply causation”^1^, but there is still tendency to confuse correlation with causation in modern scientific research. A widely known example^2^ occurred in epidemiological studies where it was found that women undergoing hormone replacement therapy (HRT) had a lower-than-average probability of incurring coronary heart disease (CHD), leading to the proposal that HRT can be effective at suppressing CHD. More carefully designed control experiments showed, however, that women who took HRT were more likely to belong to higher social-economic groups with healthier diet and regular physical exercise. In fact, the use of HRT and reduction in CHD can both be attributed to the common cause of social-economic origin, but they have no causal relation with respect to each other. To be able to correctly and accurately detect directed dynamical influence is generally of fundamental importance to many branches of science and engineering^3^,^4^ ranging from neuroscience^5^,^6^,^7^ and climatology^8^,^9^ to economics^10^,^11^,^12^.

In real world situations a precise mathematical model of the underlying system is often unavailable, thus one must rely on measured time series, data, or other types of information to uncover the directed dynamical influence in the system. Such influences generally are quite subtle - noise in the time series or uncertainties in the available information constitute a serious obstacle to successful unraveling of the influence. Conventional wisdom would stipulate that noise should be removed from data as much as possible. In this paper, however, we report a surprising phenomenon: in a recently developed, nonlinear dynamics based framework^13^, noise can counterintuitively enhance the detectability of directed dynamical influence. In fact, intentionally injecting a suitable amount of measurement noise into the time series can optimize a quantitative measure (to be described below) characterizing the detectability, which we establish using physical reasoning based on analyzing the interplay between nonlinearity and stochasticity, as well as examples from real data and model systems. Our results suggest that, in situations where ambiguity arises in the detection of directed dynamical influence injecting a certain level of noise into the time series may provide an effective resolution, leading to more reliable detection. We note that this phenomenon is distinct from stochastic resonance^14^,^15^, as noise in our case can be either additive or dynamical. In fact, the beneficial role of noise uncovered here is characteristically different because it emerges from a human designed framework/scheme to detect directed dynamical influence, one of the most subtle and elusive properties of dynamical systems.

Traditionally, causation detection is done using methods based on either the Granger causality test^16^ or transfer entropy^17^. The Granger test is a linear method operated on the hypothesis that the underlying system can be described as a multivariate stochastic process. Thus, in principle, the method is ineffective for nonlinear systems, in spite of efforts to extend the methodology to strongly coupled systems^18^,^19^,^20^. In the traditional Granger framework, measurement noise is generally detrimental in the sense that, as its amplitude is increased the value of the detected causal influence measure decreases monotonically, leading to spurious detection outcomes^21^. The transfer entropy framework is applicable^17^ to both linear and nonlinear systems, but often the required data amount is prohibitively large. In the special case of Gaussian dynamical variables, the two methods, one of the autoregressive nature (Granger test) and another based on information theoretic concepts (transfer entropy), are in fact equivalent to each other^22^. Quite recently, an alternative information theoretic measure, the causation entropy, was proposed^23^,^24^,^25^. In our study, we exploit the recent framework of convergent cross mapping (CCM)^13^ based on delay-coordinate embedding, the paradigm of nonlinear time series analysis^26^,^27^,^28^,^29^. The CCM method can deal with both linear and nonlinear systems with small data sets, and it has been applied to data from different contexts, such as EEG data^30^, FMRI^31^, fishery data^32^, economic data^33^, and cerebral auto-regulation data^34^. Here, we consider bivariate nonlinear time series from both experimental and model studies of a classic predator-prey system^13^,^35^, and investigate systematically the effects of intentionally injecting noise on detection of directed dynamical influence.

## Results

### Evidence of beneficial role of noise in detecting directed dynamical influence from an experimental data set

We consider a classic experimental prey-predator system with sustained oscillations in prey and predators, the system of *Paramecium aurelia* and *Didinium nasutum*^13^,^36^,^37^,^38^. In ref. 13, it was shown that there exists a stronger top-down control from Didinium *x* to Paramecium *y*, so *x* and *y* are the driving (predator) and driven (prey) variables, respectively, which naturally defines a directed dynamical influence. To demonstrate the beneficial role of noise in directed dynamical influence detection, we inject independent noise into the original *x* and *y* data (see Supplementary Note 1 for a description of the data set):


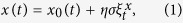



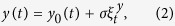


where *x*_0_(*t*) and *y*_0_(*t*) are the original time series normalized to unit mean and variance, 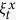 and 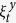 are white noise of zero mean and unit variance, *σ* is the noise amplitude, and *η* is a control parameter characterizing the *ratio of asymmetry* of the noise perturbation to the original predator and prey variables. The quality of CCM index detection can be measured^13^ by the quantity *R* (see **Methods**), where a larger positive value of *R* indicates a stronger directed dynamical influence from *x* to *y*. For the experimental system studied here, for *σ* = 0 the value of *R* is about 0.035.

**Figure 1:**
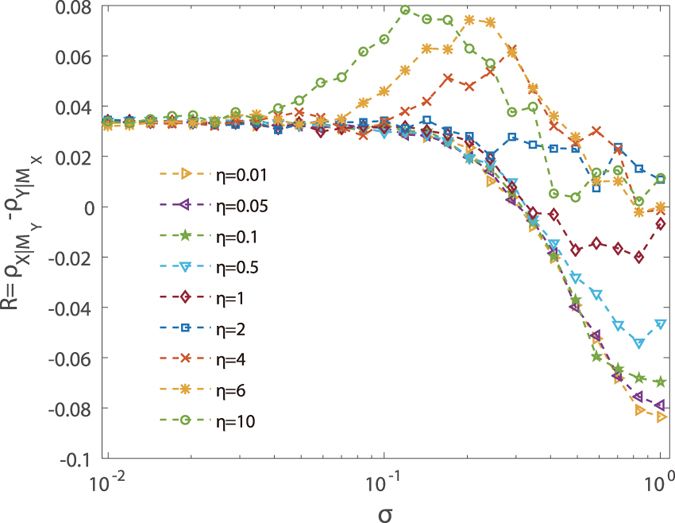
Effect of noise on detecting directed dynamical influence from an experimental data set. For the predator-prey system of *paramecium* and *Didinium*, quantity *R* characterizing the detectability versus the noise amplitude *σ*. The time series contains *L* = 61 records, into which noise is intentionally injected. The results are averaged over 1000 realizations of noise. The dimension of the reconstructed phase space is *E*_*x*_ = *E*_*y*_ = 3.

Figure 1 shows *R* versus the noise amplitude *σ* from the CCM method. We observe the phenomenon that *R* can be maximized for some optimal value of the noise amplitude when there is an asymmetry in the injected noise to the predator and prey data. As the noise amplitude *σ* is increased, *R* increases and reaches maximum. Strikingly, the maximally achievable value of *R* can be as large as 0.08 - more than 100% improvement as compared with the case of zero noise. This indicates that, when a proper amount of asymmetric noise is injected into the data set, our ability to detect directed dynamical influence can be enhanced significantly. We note that, there exists a critical value of the asymmetry ratio *η*_*c*_ that for *η* > *η*_*c*_, the enhancement phenomenon occurs and the *R* − *σ* curve exhibits a characteristic *non-monotonic* behavior. However, for *η* < *η*_*c*_, the non-monotonic behavior is lost, and no improvement in the detectability can be achieved (for too large noise amplitude *σ*, incorrect detection occurs). This provides a practically useful criterion to apply asymmetric noise: only when comparatively larger noise is injected into the *driving* variable (i.e., *x* for this experimental prey-predator system) will the *R* − *σ* curve be non-monotonic, as exemplified in Fig. 1. For a system of unknown relationship of directed dynamical influence, a non-monotonic behavior of *R* is indication that the correct the relationship has been detected. In addition, as shown in Fig. 1, small values of the asymmetry ratio and relatively large noise amplitude can lead to incorrect assessment of the directed dynamical influence^13^.

### Beneficial role of noise in detecting directed dynamical influence from model data set

**Figure 2:**
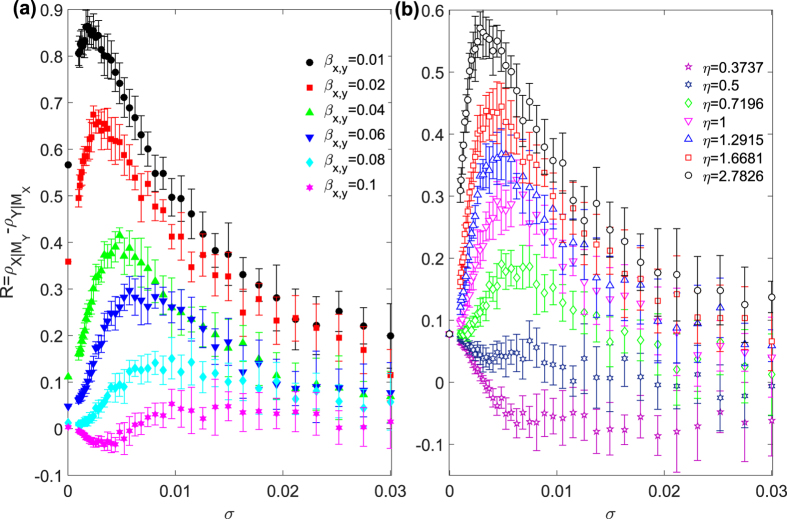
Enhancement of detectability of directed dynamical influence from model. Measure of detectability *R* versus noise amplitude *σ* for a model ecosystem: (**a**) for different values of *β*_*x*,*y*_ but fixed *β*_*y*,*x*_ = 0.1 and *η* = 1, (**b**) for different values of the asymmetry parameter *η* but for fixed *β*_*x*,*y*_ = 0.05 and *β*_*y*,*x*_ = 0.1. The value of *R* for each parameter setting is averaged over 10 dynamical realizations and 10 different noise arrangements for each dynamical realization. Other parameters are *r*_*x*_ = 3.8 and *r*_*y*_ = 3.5.

In general, noise can cause two relevant quantities, 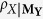 and 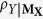, to decay, where 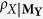 is the CCM measure from *X* to *Y*, with larger value indicating a higher casual effect of the variable *x* to the variable *y*, and 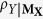 has a similar meaning. (The detailed definitions of 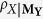 and 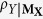 are given in **Methods**.) The key point is that, due to the directed dynamical influence, the *decay rates* are different, so the difference *R* between 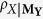 and 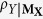 can be maximized for proper amount of noise, leading to enhancement of detectability. To develop a physical understanding of the counterintuitive phenomenon in a concrete setting and also to be able to compare results directly with those from real data in Fig. 1, we consider a two-dimensional ecosystem model^13^:


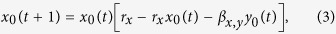



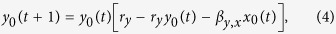


where *r*_*x*_, *r*_*y*_ ∈ [0, 4] are parameters of the intrinsic population dynamics, *β*_*x*,*y*_ ∈ [0, 0.1] and *β*_*y*,*x*_ ∈ [0, 0.1] are the coupling parameters from *y*_0_ to *x*_0_ and vice versa, respectively. The degree of directed dynamical interaction between the two variables can be adjusted by changing the relative values of the coupling parameters. As in our analysis of the experimental data, we inject measurement noise into the original time series *x*_0_ and *y*_0_ to obtain the corresponding new time series *x* and *y*. The dimensions of the reconstructed phase space for *x* and *y* are *E*_*x*_ = *E*_*y*_ = 2, and the length of the time series is *L* = 1001. We choose *β*_*x*,*y*_ < *β*_*y*,*x*_ so that the *x*-dynamics has a stronger influence on the *y*-dynamics than that in the opposite direction, indicating a larger directed dynamical influence from *x* to *y*. Indeed, we obtain from numerics that the value of *R* is positive and increases with the difference (*β*_*y*,*x*_ − *β*_*x*,*y*_). Figure 2(a) shows *R* versus the noise amplitude *σ* for an increasing set of *β*_*x*,*y*_ values (from 0.01 to 0.1) but for fixed *β*_*y*,*x*_ = 0.1. We see that *R* can be maximized by noise (for *σ* ≈ 0.005), similar to the behavior observed from the experimental data set (Fig. 1). We also validate that, when coupling between the two dynamical variables is symmetric, i.e., for *β*_*x*,*y*_ = *β*_*y*,*x*_ = 0.1, the value of *R* tends to fluctuate about zero as *σ* is increased, as it should be. Both the non-monotonic behavior of *R* and the overall increase in the value of *R* with *η* can be attributed to the more rapid decay of the correlation coefficient 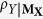 associated with larger *η* (see Supplementary Note 2 for additional examples and Figs S1 and S2 in Supplementary Information for the detailed behaviors of the correlation coefficients 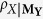 and 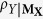). Figure 2(b) compares the results from different values of noise asymmetry ratio *η* for fixed dynamical coupling (*β*_*x*,*y*_ = 0.05 and *β*_*y*,*x*_ = 0.1). We see that, as *η* is increased, the overall curve of *R* versus *σ* is elevated, and the position of the peak moves toward the region of smaller *σ* values.

**Figure 3:**
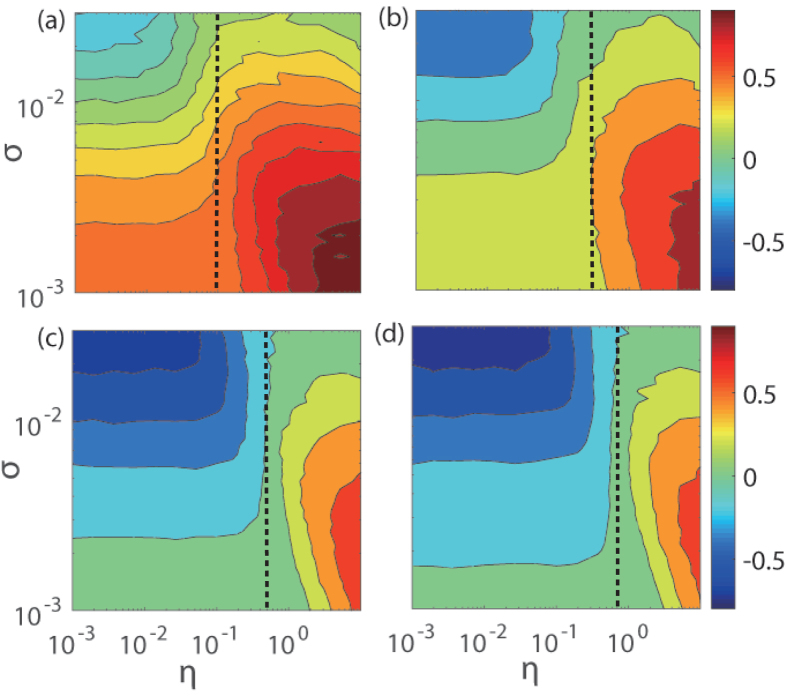
Measure of detectability *R* in the parameter plane of noise. The parameters characterizing the properties of measurement noise are *σ* and *η*. The model system has fixed *β*_*y*,*x*_ = 0.1 and different values of *β*_*x*,*y*_: (**a**) 0.01, (**b**) 0.02, (**c**) 0.05, and (**d**) 0.07. Other parameters are *r*_*x*_ = 3.8, *r*_*y*_ = 3.5 and *L* = 1001. The values of the threshold *η*_*c*_ = *β*_*x*,*y*_/*β*_*y*,*x*_ above which noise enhancement of CCM index measure occurs are marked by the dashed lines.

To obtain a comprehensive understanding of the role of noise in detecting the directed dynamical influence, we calculate *R* for the whole parameter plane (*η*,*σ*) characterizing the properties of measurement noise for *β*_*y*,*x*_ > *β*_*x*,*y*_, as shown in Fig. 3. We see that the noise enhancement effect takes place in the region of *η* > *η*_*c*_ (for *η* < *η*_*c*_, *R* decreases monotonically with the noise amplitude). In each panel, the value of *η*_*c*_ is indicated by a dashed line. We also note that, in the upper-left region of the parameter plane (i.e., small *η* and large *σ*), the values of *R* are negative, indicating incorrect identification of directed dynamical influence. This can be explained from the dynamical structures in the reconstructed phase space. In particular, for the case of extremely small values of *η*, noise in *x* makes the value of *D*_*x*_ = *ησ* too small to induce a significant decay of 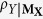 with *σ*. However, 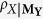 decays to a smaller value (e.g., for *η* = 0.3727 in Fig. 2), leading to negative values of *R*. For the case where bidirectional causation is more homogeneous, i.e., *β*_*y*,*x*_ and *β*_*x*,*y*_ have approximately the same values, as shown in Fig. 3(a–d), the region of incorrect CCM index detection is enlarged. All these indicate that the applicability of the CCM method depends on how noise is introduced into the measured time series. When this is done properly, detectability of directed dynamical influence can be greatly enhanced.

### Physical theory

**Figure 4:**
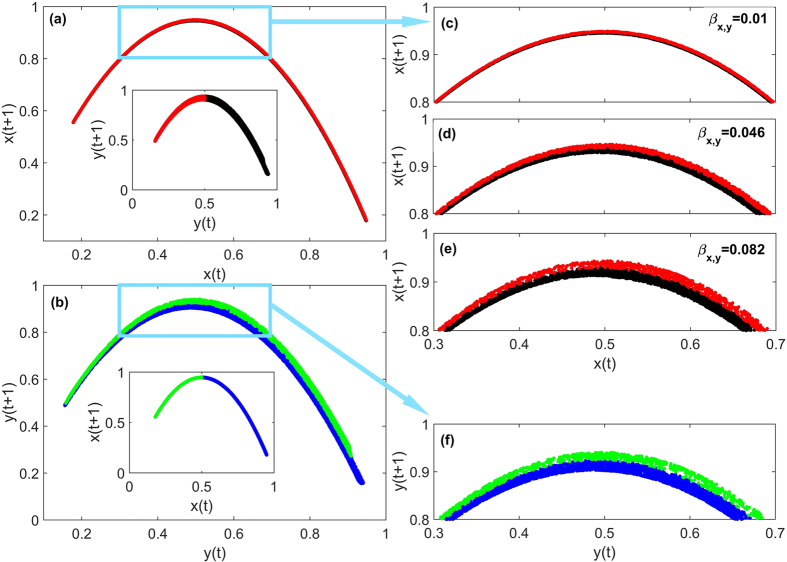
Effect of noise on reconstructed phase space of dynamical variables. Attractor manifolds **M**_**X**_ (**a,c–e**) and **M**_**Y**_ (**b,f**) for *σ* = 0 and *β*_*y*,*x*_ = 0.1. The insets in (**a,b**) show the manifold counterparts, respectively, with the color scheme indicated. For example, in (**a**), the dots (*x*(*t*), *x*(*t* + 1)) in **M**_**X**_ is colored according to the corresponding point *y*(*t*) in **M**_**Y**_, where points with *y*(*t*) ≥ 0.5 are in black and those with *y*(*t*) < 0.5 in red. The values of *β*_*x*,*y*_ are 0.01 (**a,c**), 0.046 (**d**), and 0.082 (**e**), respectively. Panels (**c,f**) are magnifications of the squared frames in (**a,b**), respectively. Other parameters are *r*_*x*_ = *r*_*y*_ = 3.8.

An effective approach to gaining a physical understanding of the role of noise in promoting directed dynamical influence detection is through examination of the relative effects of coupling and noise on “disturbing” the phase space structure. In general, the dynamics of the system is determined by the structure of the attractor manifold. In the model system, there is an influence of *y*_0_ on *x*_0_ through the coupling term −*β*_*x*,*y*_ *x*_0_(*t*)*y*_0_(*t*). In this sense, we say that *y*_0_ has an effect on the ordered structure of the manifold **M**_**X**_, where **M**_**X**_ is the shadow manifold constructed from the time series *x*_0_ with embedding dimension *E*_*x*_ and time-delay *τ*. Similarly, **M**_**Y**_ is the shadow manifold obtained from the time series *y*_0_ with the same parameter (see a detailed explanation in **Methods**). For a given value of *x*_0_(*t*), the points (*x*_0_(*t*), *x*_0_(*t* + 1)) in **M**_**X**_ spread in a region with its upper and lower bounds determined by the possible minimum and maximum values of *y*_0_, respectively. The effect of *y*_0_ on **M**_**X**_ can be characterized by the width in the reconstructed phase space:


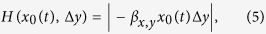


where Δ*y* = max(*y*_0_) − min(*y*_0_). More specifically, the detailed behavior of *y*_0_ contributes to the formation of the structure of **M**_**X**_. For example, a continuously distributed *y*_0_ (e.g., for *r*_*y*_ = 3.8) leads to a continuous strip of **M**_**X**_, as shown in Fig. 4, but two separated clusters of *y*_0_ (e.g., for *r*_*y*_ = 3.5) produces a pair of long parallel strips in **M**_**X**_ (see Fig. S3 in Supplementary Information).

To better understand the mutual influence between **M**_**X**_ and **M**_**Y**_, we color the dots in one state space according to their corresponding positions in the counterpart state space. Take the case shown in Fig. 4(a) as an example. We select a boundary in the middle of the manifold **M**_**Y**_ [e.g., *y*(*t*) = 0.5, as shown in the inset of panel (a)] and color the dots on the left-hand and right-hand sides in red and black, respectively, where each dot in **M**_**Y**_ corresponds to one point (*y*(*t*), *y*(*t* + 1)) for certain value of *t*. We set the same color to the dot (*x*(*t*), *x*(*t* + 1)) in the manifold **M**_**X**_. This color scheme presents us with a clear picture of the structural relationship between the manifolds **M**_**X**_ and **M**_**Y**_. For example, for the two clusters in **M**_**Y**_ (denoted by the value 0.5), the corresponding structure in **M**_**X**_ consists of two stretched, thin strips that are close to each other. The dynamics on the two thin strips in **M**_**X**_ are sensitive to noise, since a small perturbation can move a dot from one strip to another, visually leading to a mixing of dots of different colors. As we estimate 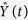 according to the dots in **M**_**X**_ through the neighbors of *X*(*t*), noise of amplitude about the thickness of the strips will result in a decrease in the prediction accuracy. The thickness of the strip in **M**_**X**_ depends on the dynamical coupling from *y*. For the extreme case of *β*_*x*,*y*_ = 0, i.e., *y* is decoupled from *x*, the thickness of the strip in **M**_**X**_ becomes zero. As *β*_*x*,*y*_ is increased, the strip in **M**_**X**_ becomes thicker, as shown in Fig. 4(c–e).

The features of *y*_0_ thus determine how the structure of the manifold **M**_**X**_ is modified through the coupling parameter *β*_*x*,*y*_. A relatively small value of *β*_*x*,*y*_ indicates a weaker influence from *y*_0_ to *x*_0_, which results in a narrower strip in **M**_**X**_, and some larger value of *β*_*x*,*y*_ will enlarge the width *H*(*x*_0_, Δ*y*) of **M**_**X**_, as shown in Fig. 4(c–e). To illustrate the influence of *y*_0_ on *x*_0_, we mark the points in **M**_**Y**_ with *y*_0_(*t*) < 0.5 and *y*_0_(*t*) ≥ 0.5 with red and black colors, respectively. The corresponding points in **M**_**X**_ at the same instants are marked by the same color. We see that the points in **M**_**X**_ corresponding to different values of *y*_0_(*t*) tend to spread farther away from each other, and **M**_**X**_ exhibits separated long pairing strips. Analogously, the effect of *x*_0_ on *y*_0_ is embedded in the structure of **M**_**Y**_ of size determined by


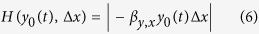


and Δ*x* = max(*x*_0_) − min(*x*_0_). The widths of **M**_**X**_ and **M**_**Y**_, as determined by the coupling between the two variables, play a crucial role in system’s response to noise associated with the detection of directed dynamical influence.

The phenomenon of noise enhanced detection of directed dynamical influence can be intuitively understood in terms of noise-induced diffusion. In particular, in the reconstructed phase space, noise can be viewed as inducing random diffusion of points on the attractor manifolds, which alters the originally ordered phase space structures that are result of the mutual dynamical interactions. On the manifold **M**_**X**_, what is relevant is the expected diffusion radius 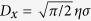 of point *X*(*t*), with respect to the width *H*(*x*(*t*),Δ*y*) of the manifold due to the influence from *y*_0_. In predicting *Y*(*t*) according to its weighted average 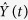, the *E* + 1 nearest neighbors of *X*(*t*), denoted as *X*(*t*_*i*_) in **M**_**X**_, are taken into account. However, diffusion can introduce “wrong” neighboring points from the region within the average distance *D*_*x*_. When these points are mapped to **M**_**Y**_, they correspond to the points *Y*(*t*_*i*_) with horizontal ordinate *y*(*t*) deviating by the amount


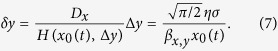


The points in **M**_**Y**_ with the horizontal ordinates *y*(*t*) ± *δy* are actually used to estimate 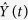 (see Fig. S3 in Supplementary Information for a specific example). Here, *δy* is a measure of how errors in *x*(*t*) (due to noise) propagate to the corresponding errors in *y*(*t*). The accuracy of the estimation *Y*(*t*) decreases with *δy*. For the normalized time series (i.e., 〈*x*_0_〉 = 1), the average deviation is given by


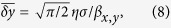


where a smaller effective noise amplitude *ησ* on *x*_0_ or a larger coupling parameter *β*_*x*,*y*_ from *y*_0_ to *x*_0_ will yield a smaller value of 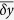, leading to more accurate estimation 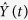. The quantity 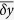 thus characterizes the competition between the effects on *y*_0_ from *x*_0_ (denominator) and that from noise (numerator). A similar picture arises when estimating 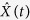 based on **M**_**Y**_, where the width of the manifold **M**_**Y**_ is *H*(*y*_0_(*t*), Δ*x*), the expected vertical diffusion radius is 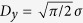, and the corresponding points *X*(*t*_*i*_) for the weighted average have the deviation


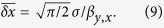


We thus see that, interaction from the coupled variable contributes to forming a wider well-organized manifold of the focal variable, while noise tends to destroy the order of the structure. The competition between nonlinear interaction and noise in detecting directed dynamical influence is general and independent of the system and measurement details.

Consider the setting where the variable *x* is the cause to *y*, as for *β*_*y*,*x*_ > *β*_*x*,*y*_ in the model system. The distinct responses of *x* and *y* to noise can lead to enhancement of *R*. Our analysis suggests 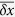 and 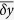 as the indicators of the competition between coupling and noise. The ratio


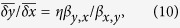


quantifies the relative decay rate of 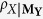 with respect to 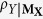 as the noise amplitude *σ* is increased. A faster decay of 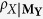 to zero with *σ*, which in CCM prediction is associated with 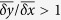, leads to the non-monotonic behavior in *R* versus *σ*. Furthermore, the condition 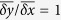 defines the threshold ratio *η*_*c*_ (or critical value) for the emergence of the non-monotonic behavior (Fig. 3). For a real system with the directed dynamical relationship unknown *a priori*, the ratio *β*_*x*,*y*_/*β*_*y*,*x*_ can be estimated by injecting asymmetric noise into the time series and calculating the threshold *η*_*c*_. As shown in Fig. 1, the experimental system has the threshold *η*_*c*_ around 1.5. We also find that, for systems under dynamical noise, detection of directed dynamical influence can still be enhanced, as described in Supplementary Note 3 and shown in Fig. S4.

## Discussions

We have uncovered a practically implementable mechanism to significantly enhance detection of directed dynamical influence in nonlinear dynamical systems: injecting asymmetric noise into the time series of the dynamical variables of interest. The general idea is that, since directed dynamical influence reflects the differential interaction of the dynamical variables on each other, noise can lead to an *asymmetric* degradation of the interactions. Because of the nonlinear dynamical underpinning of the CCM algorithm, its performance can in fact be enhanced and optimized by noise, which is the main result of our work. In particular, the CCM method relies on the asymmetry in the directed dynamical influence measures between the two dynamical variables in the two opposite directions. When noise of non-identical amplitude is added to the two variables, the asymmetry in the directed dynamical influence measures can be amplified, leading to better performance in the detection. For example, let *x* and *y* be the two dynamical variables, and assume that the directed dynamical influence from *x* to *y* is stronger than that for the opposite direction: *β*_*y*,*x*_ > *β*_*x*,*y*_. As the noise amplitude is increased to a level corresponding to the weaker directed dynamical influence from *y* to *x* as characterized by *β*_*x*,*y*_ but not yet up to that from *x* to *y* (characterized by *β*_*y*,*x*_), the ability to predict *y* from *x* will be dramatically reduced but that in the opposite direction will be affected less. As a result, the difference (*β*_*y*,*x*_ − *β*_*x*,*y*_) will be enhanced. In other words, the beneficial role of noise can be attributed to the fact that weaker dynamical influence is destroyed earlier than the stronger one as the noise amplitude is increased. However, if the noise amplitude reaches the level of the stronger directed dynamical influence from *x* to *y*, the algorithm will not be able to detect any such influence. As a result, a non-monotonic relation between the detectability of directed dynamical influence and noise amplitude arises, in contrast to the monotonic decreasing behavior associated with the original Granger method.

It should be noted that, while a larger value of the metric *R* suggests a larger degree of asymmetry between the directed dynamical influence of dynamical variables in the two opposite directions, the task of enhancing the value of *R* should not be confused with that of detecting causality in the first place. Often, for any pair of variables in a nonlinear dynamical system, there is typically directed dynamical influence in both directions. That is, the question of whether there is causality has a trivial answer. The nontrivial and challenging task is to assess which direction possesses a stronger directed dynamical influence. Our main finding is that an appropriate amount of asymmetric noise can facilitate the assessment.

Intuitively, the phenomenon of noise enhanced detection of directed dynamical influence can be understood by resorting to the picture of noise induced diffusion in the phase space. The explanation is heuristic, calling for a quantitative or even rigorous analysis, which remains to be an outstanding issue at the present. Practically, the phenomenon noise can be induced *extremely readily* utilizing time series only, and we expect it to be appealing to ascertaining directed dynamical influence in experiments or data analysis of complex dynamical systems.

We remark that, while the intuitive argument that noise disrupts the structure of the noise-free manifold of the dynamical system is reasonable, it may not be generally true that the magnitude of the error can be preserved as measured by the directed dynamical influence metric *R*. For the systems studied in this paper, the coupling functions between the dynamical variables are assumed to be linear, i.e., the effect of one variable on the other is linear. This, however, may not be true generally. In particular, if the response of one variable to the other is nonlinear, small errors could be amplified and large errors could be reduced, and the nonlinear amplification/reduction effect can be state-dependent. In realistic systems there is no guarantee that the response of one variable to another is even a continuous function. For such cases, the effect of noise on detecting directed dynamical influences would depend on the system details. For complex dynamical systems with nonlinear or discontinuous coupling functions, whether a general relation exists between a measure of directed dynamical influence and the noise strength is an open question deserving further investigation.

## Methods

### CCM method

The nonlinear-dynamics based method was proposed recently^13^ to detect and quantify directed dynamical influence between a pair of dynamical variables through the corresponding time series. The starting point is to reconstruct a phase space, for each variable, based on the delay-coordinate embedding method^26^. Specifically, for time series *x*(*t*), the reconstructed vector is *X*(*t*) = [*x*(*t*), *x*(*t* − *τ*), …, *x*(*t* − (*E*_*x*_ − 1)*τ*)], where *τ* is the delay time and *E*_*x*_ is the embedding dimension. For variable *y*, a similar vector can be constructed in the *E*_*y*_ dimensional space. Let **M**_**X**_ and **M**_**Y**_ denote the attractor manifold in the *E*_*x*_- and *E*_*y*_-dimensional space, respectively. If *x* and *y* are dynamically coupled, there is a mapping relation between **M**_**X**_ and **M**_**Y**_. The CCM method measures how well the local neighborhoods in **M**_**X**_ correspond to those in **M**_**Y**_. In particular, the cross-mapping estimate of a given *Y*(*t*), denoted as 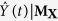, is based on a simplex projection^39^,^40^ that is essentially a *nearest-neighbor algorithm* involving *E* + 1 nearest neighbors of *X*(*t*) in **M**_**X**_. (Note that *E* + 1 is the minimum number of points required for a bounding simplex in the *E*-dimensional space.) The time indices of the *E* + 1 nearest neighbors are denoted as *t*_1_, *t*_2_, …, *t*_*E*+1_ in the order of distances to *X*(*t*) from the nearest to the farthest, i.e., point *X*(*t*_1_) is the nearest-neighboring point of *X*(*t*) in **M**_**X**_. These time indices are used to identify the points (putative neighborhoods) in **M**_**Y**_, namely, to find the points at the corresponding instants: *Y*(*t*_1_), *Y*(*t*_2_), …, and *Y*(*t*_*E*+1_), which are used to estimate 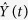 through the weighted average


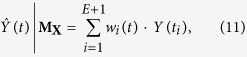


where


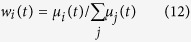


is the weight of the vector *Y*(*t*_*i*_),


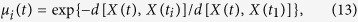


and *d*[*X*(*t*), *X*(*t*_*i*_)] is the Euclidean distance between the two vector points *X*(*t*) and *X*(*t*_*i*_) in **M**_**X**_. An estimated time series 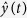 can then be obtained from 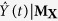. Likewise, the cross mapping from *Y* to *X* can be defined analogously so that the time series of *x*(*t*) can be predicted from the cross-mapping estimate 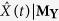.

The correlation coefficient between the original time series *y*(*t*) and the predicted time series 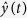 from **M**_**X**_, denoted as 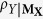, is a measure of CCM directed dynamical influence from *y* to *x*. Larger value of 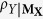 implies that *y* is a stronger cause of *x*, while 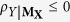 indicates that *y* has no influence on *x*. The relative strength of directed dynamical influence can be defined as 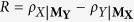, which is a quantitative measure of the casual relationship between *x* and *y*. A positive value of *R* indicates that *x* is the CCM cause of *y*. The measure *R* is used in Fig. 1 to quantify the degree of noise enhancement of CCM index detection.

### Time delay and embedding dimension

The CCM method for detecting directed dynamical influence is derived from the standard delay-coordinate embedding method^26^ in nonlinear time series analysis. For properly chosen time delay^41^ (denoted as *τ*) and embedding dimension (denoted as *E*), the phase space of the underlying dynamical system can be faithfully reconstructed from time series. There are various methods for choosing the parameters^42^ *τ* and *E*, such as those based on the mutual information^43^, the correlation integral and dimension^44^,^45^,^46^,^47^,^48^,^49^, false nearest neighbors (FNN)^50^, and nonlinear prediction criteria^51^,^52^,^53^.

In the text, the time delay and embedding dimension for the experimental predator-prey data are chosen to be *τ* = 1 and *E*_*x*_ = *E*_*y*_ = 3, respectively. For the model systems with measurement noise and dynamical noise, we choose *E*_*x*_ = *E*_*y*_ = 2 and *τ* = 1. A comprehensive treatment of the effect of noise on phase space reconstruction can be found in ref. 53.

## Additional Information

**How to cite this article**: Jiang, J.-J. *et al*. Directed dynamical influence is more detectable with noise. *Sci. Rep*. **6**, 24088; doi: 10.1038/srep24088 (2016).

## Supplementary Material

Supplementary Information
