# Supplementary material for: Directed dynamical influence is more detectable with noise
Source: Sci Rep. 2016 Apr 12;6:24088. doi: 10.1038/srep24088 (PMC4828632; doi:10.1038/srep24088)
Supplement: Supplementary Information [file srep24088-s1.pdf]

Supplementary Information for

Directed dynamical influence is more detectable with noise

Jun-Jie Jiang, Zi-Gang Huang, Liang Huang, Huan Liu, and Ying-Cheng Lai

Supplementary Note 1: Description of experimental data set

The experimental data set studied in the main text came from records of the Didinium-Paramecium protozoan prey-predator system, which is the same data set studied in Ref. [1]. The detailed experimental conditions and related historical studies [2, 3, 4] are described in the Supplemental Materials of Ref. [1]. The data can be found in Ref. [5] and downloaded from the web site: *http://robjhyndman.com/ tsdldata/data/veilleux.dat*.

The length of the data set is $L = 71$, and we remove the first 10 data points to eliminate transient behavior. For this top-down control system, the variables for the predator (Didinium) and the prey (Paramecium) are denoted as x and y, respectively. The stronger driving variable is x. The time series is normalized to having unit mean and variance.

Supplementary Note 2: Effect of measurement noise on detecting directed dynamical influence - additional examples

To better understand the behavior of the measure R in the CCM framework, we show in Figs. S1 and S2 R and the corresponding correlation coefficients, $\rho_{X|\mathbf{M_Y}}$ and $\rho_{Y|\mathbf{M_X}}$, versus the noise amplitude σ. Note that, Fig. 2(a) in the main text shows R versus σ but under a different parameter setting ($r_x = 3.5$ and $r_y = 3.8$). Here, the results are for $r_x = r_y = 3.8$ in Fig. S1, where panel (a) plots R versus σ and panel (b) shows how the corresponding correlation coefficients decay with σ. Similar to the phenomenon described in the main text, for a given value of σ, a smaller value of $\beta_{x,y}$ as compared with that of $\beta_{y,x}$ results in a larger value of R, due to the faster decay of the correlation coefficient$\rho_{Y|\mathbf{M_X}}$. From Fig. 3 in the main text, representative structures of the attractor manifold of the system for $r_x = r_y = 3.8$, we see that the faster decay in $\rho_{Y|\mathbf{M_X}}$ can be attributed to the narrower structure of $\mathbf{M_X}$, as it can be relatively readily disturbed by noise. For the symmetric case of $\beta_{x,y} = \beta_{y,x}$, the value of R fluctuates about zero as σ is increased.

Figures S2(a) and S2(b), respectively, plot R and the corresponding correlation coefficients $\rho_{X|\mathbf{M_Y}}$ and $\rho_{Y|\mathbf{M_X}}$ versus σ for different values of the asymmetric noise ratio η. The parameters are the same as Fig. 2(b) in the main text ($r_x = 3.8$, $r_y = 3.5$, $\beta_{x,y} = 0.05$, and $\beta_{y,x} = 0.1$). Since $\beta_{y,x} > \beta_{x,y}$, x_0 affects y_0 more than the other way around, and a successful detection of directed

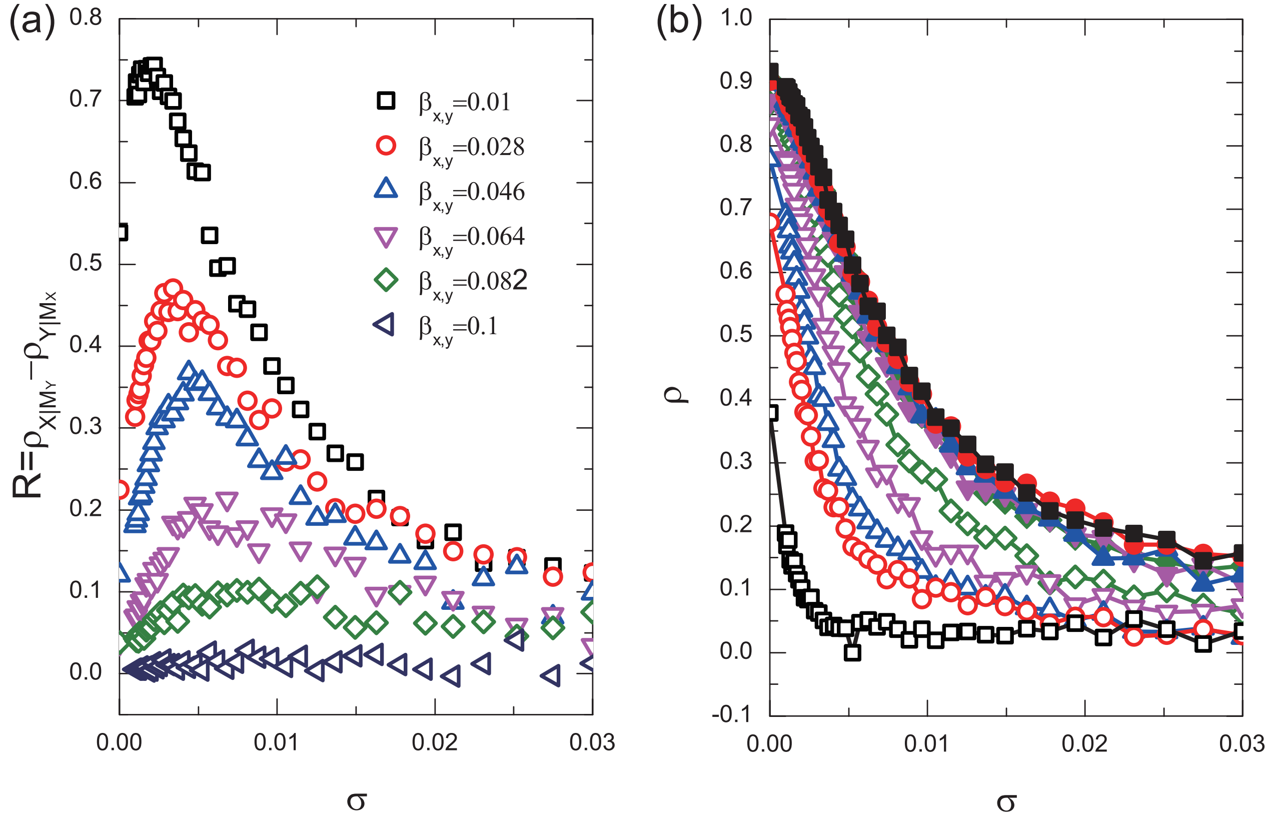

Supplementary Figure S1 | For CCM analysis of the predator-prey model system described in the main text, (a) measure R as a function of the noise amplitude σ for different values of $\beta_{x,y}$ and (b) the corresponding correlation coefficients $\rho_{X|\mathbf{M_Y}}$ (closed symbols) and $\rho_{Y|\mathbf{M_X}}$ (open symbols). For (a) and (b), the values of $\beta_{x,y}$ are 0.01 (squares), 0.028 (circles), 0.046 (up triangles), 0.064 (down triangles), 0.082 (diamond), 0.1 (left triangles). Other parameters are $\eta = 1$, $\beta_{y,x} = 0.1$, and $r_x = r_y = 3.8$.

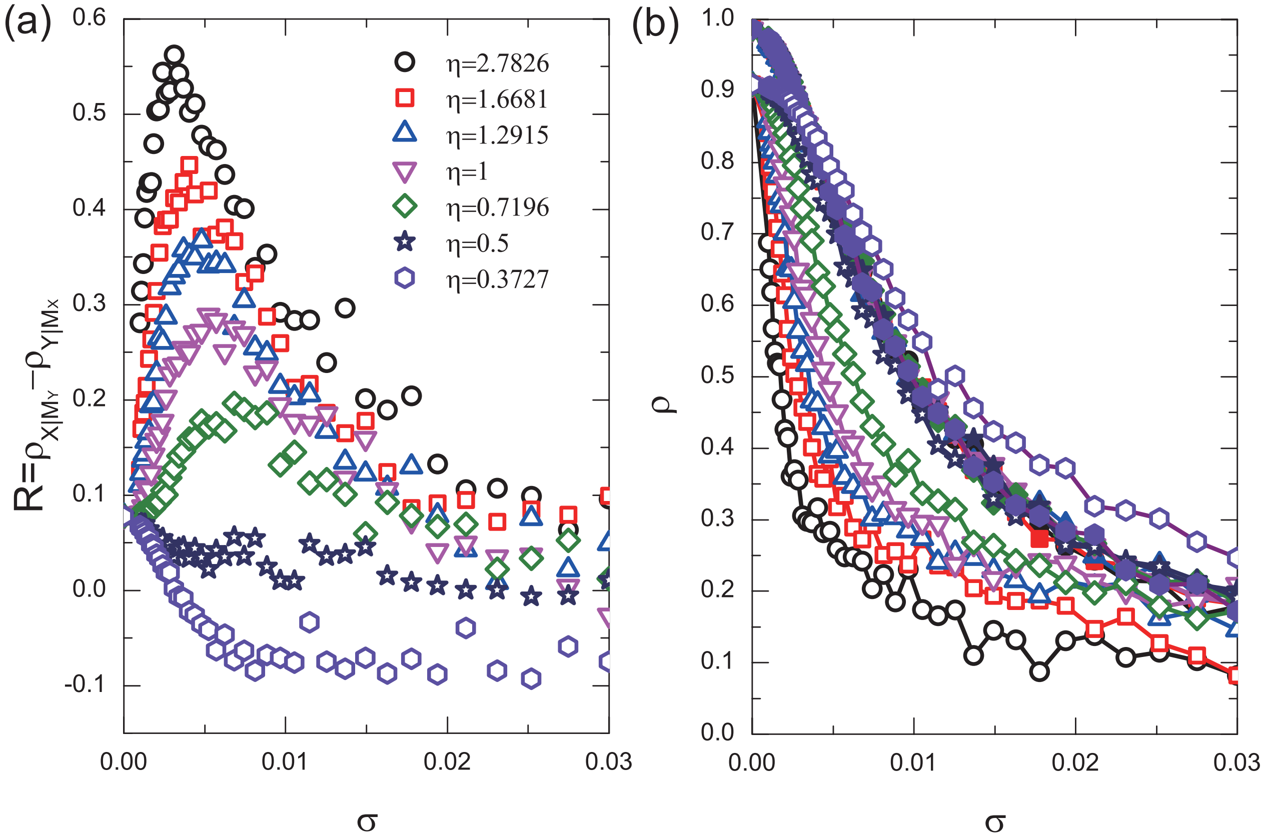

Supplementary Figure S2 | For CCM analysis of the model predator-prey system in the main text, (a) measure R as a function of the noise amplitude σ for different values of the asymmetry ratio η, and (b) the corresponding correlation coefficients $\rho_{X|\mathbf{M_Y}}$ (closed symbols) and $\rho_{Y|\mathbf{M_X}}$ (open symbols). The values of η are 2.7826 (circles), 1.6681 (squares), 1.2915 (up triangles), 1.0 (down triangles), 0.7196 (diamond), 0.5 (pentagram), and 0.3727 (hexagon). Other parameters are $\beta_{x,y} = 0.05$, $\beta_{y,x} = 0.1$, $r_x = 3.8$, and $r_y = 3.5$.

dynamical influence would yield positive values for R. As shown in Fig. S2(a) [or Fig. 2(b) in the main text], in absence of noise ($\sigma = 0$), the value of R is about 0.0778, reflecting the directed dynamical relationship correctly. As measurement noise is introduced, the value of R increases with σ first, reaches a maximum, and then decreases, and this type of non-monotonic behavior occurs for $\eta > \eta_c = \beta_{x,y}/\beta_{y,x}$. As shown in Fig. S2(b), both $\rho_{X|\mathbf{M_Y}}$ and $\rho_{Y|\mathbf{M_X}}$ decay with σ, where $\rho_{X|\mathbf{M_Y}}$ is approximately independent of the value of η but $\rho_{Y|\mathbf{M_X}}$ depends sensitively on η. As η is increased, the decay rate of the correlation coefficient $\rho_{Y|\mathbf{M_X}}$ in the small σ region increases. Additionally, for a given value of σ, a larger value of η corresponds to larger effective noise on $x(t)$, resulting in a larger value for the ratio $\overline{\delta y}/\overline{\delta x}$. As a result, $\rho_{Y|\mathbf{M_X}}$ decays more rapidly, as shown in Fig. S2(b). In fact, for large values of η (beyond η_c), both the non-monotonic behavior of R and the overall increase of R with η observed in panel (a) can be attributed to the faster decay of the correlation coefficient $\rho_{Y|\mathbf{M_X}}$.

Figure S3 shows the reconstructed attractor manifolds of x and y for the two cases where there is no intentional noise (a,b) and there is noise of amplitude $\sigma = 0.005$ (c,d). The parameters of the system are set to be $r_x = 3.8$ and $r_y = 3.5$ so that, if the x and y variables are decoupled, there is a chaotic attractor for x and a two-piece attractor for y. The attractor manifold of y consists of two small, separated clusters [panel (b)]. For better visualization, we mark the two clusters in $\mathbf{M_Y}$ with black and red (gray) colors, and colored the corresponding points (at the same instants of time) in $\mathbf{M_X}$ accordingly. We see that attractor manifold of x exhibits a pair of long curves close to each other [panel (a)]. When a small amount of measurement noise is intentionally injected into the time series, as shown in panel (c), the red (gray) and black clusters in $\mathbf{M_X}$ merge at the boundary, impacting the accuracy of cross mapping estimation of $Y(t)$ based on $\mathbf{M_X}$, i.e., $\hat{Y}(t)|\mathbf{M_X}$. In particular, in the merged region of the two clusters, as result of noise, a red (gray) point $X(t')$ in the black cluster [marked by open circle pointed to by a red thick arrow in the inset of Fig. S3(c)] may be selected as the neighbor of a black point $X(t)$ (indicated by the black thick dashed arrow in the inset). The point $X(t')$ is thus an incorrect neighboring point. Consequently, point $Y(t')$ in the red (gray) cluster of $\mathbf{M_Y}$ [marked by open circle pointed to by a red thick arrow in Fig. S3(d)] corresponding to $X(t')$ will be used to estimate the position of the point $Y(t)$ (indicated by a black thick dashed arrow). We see that $Y(t')$ is in fact far away from the black cluster of $\mathbf{M_Y}$ where the actual point $Y(t)$ is located. As the number of incorrectly estimated neighboring points is increased due to noise-induced merging of the parallel clusters in $\mathbf{M_X}$, the accuracy of the estimated $Y(t)$ will decay dramatically. (The two correct neighboring points of $X(t)$ and the two corresponding cross mapping points in $\mathbf{M_X}$ are indicated by thin black arrows.)

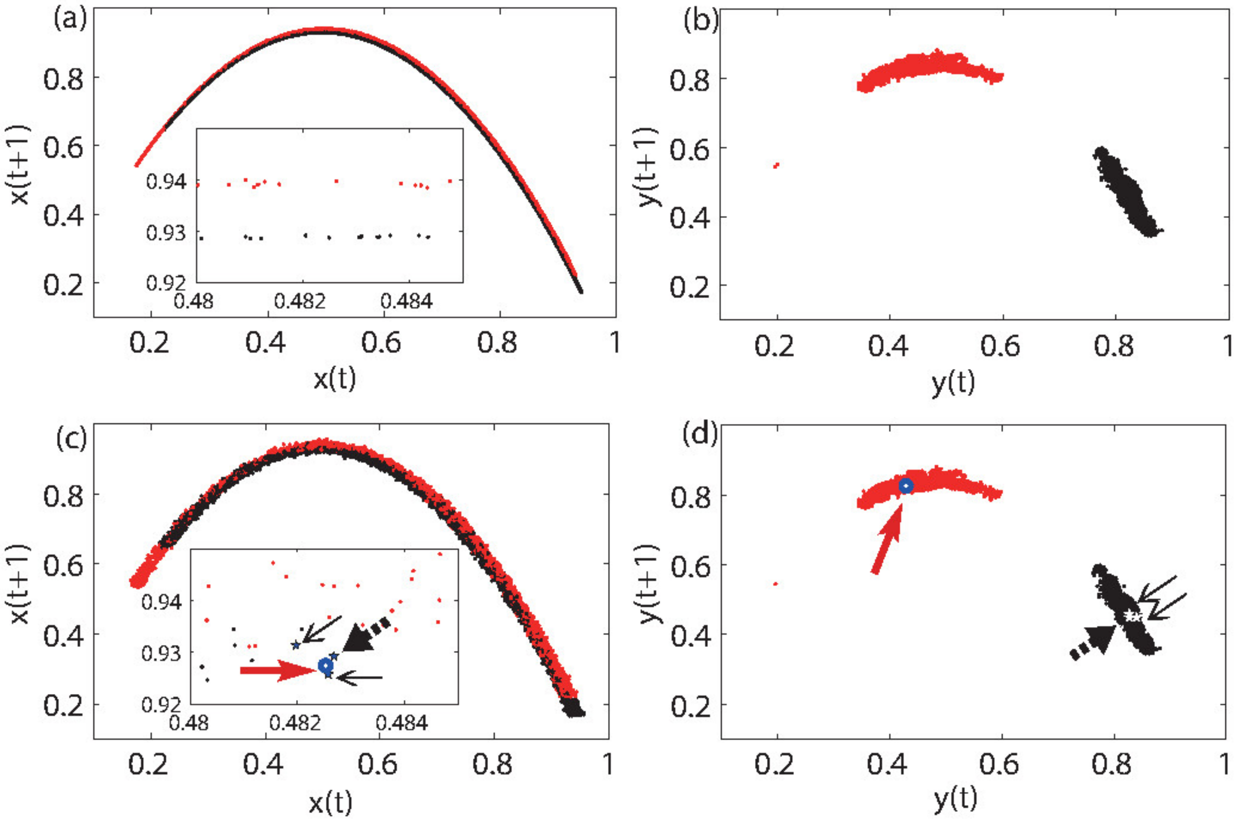

Supplementary Figure S3 | For the model predator-prey system treated in the main text, reconstructed phase space for $\hat{Y}(t)|\mathbf{M_X}$: attractor manifolds of x and y for $\sigma = 0$ (a,b) and $\sigma = 0.005$ (c,d). The two separated clusters of points in the phase space in panels (b,d) are labeled with red (gray) and black colors, respectively, and the corresponding points (i.e, at the same time instant t) in (a,c) are similarly colored in (b,d). Other parameters are $r_x = 3.8$ and $r_y = 3.5$.

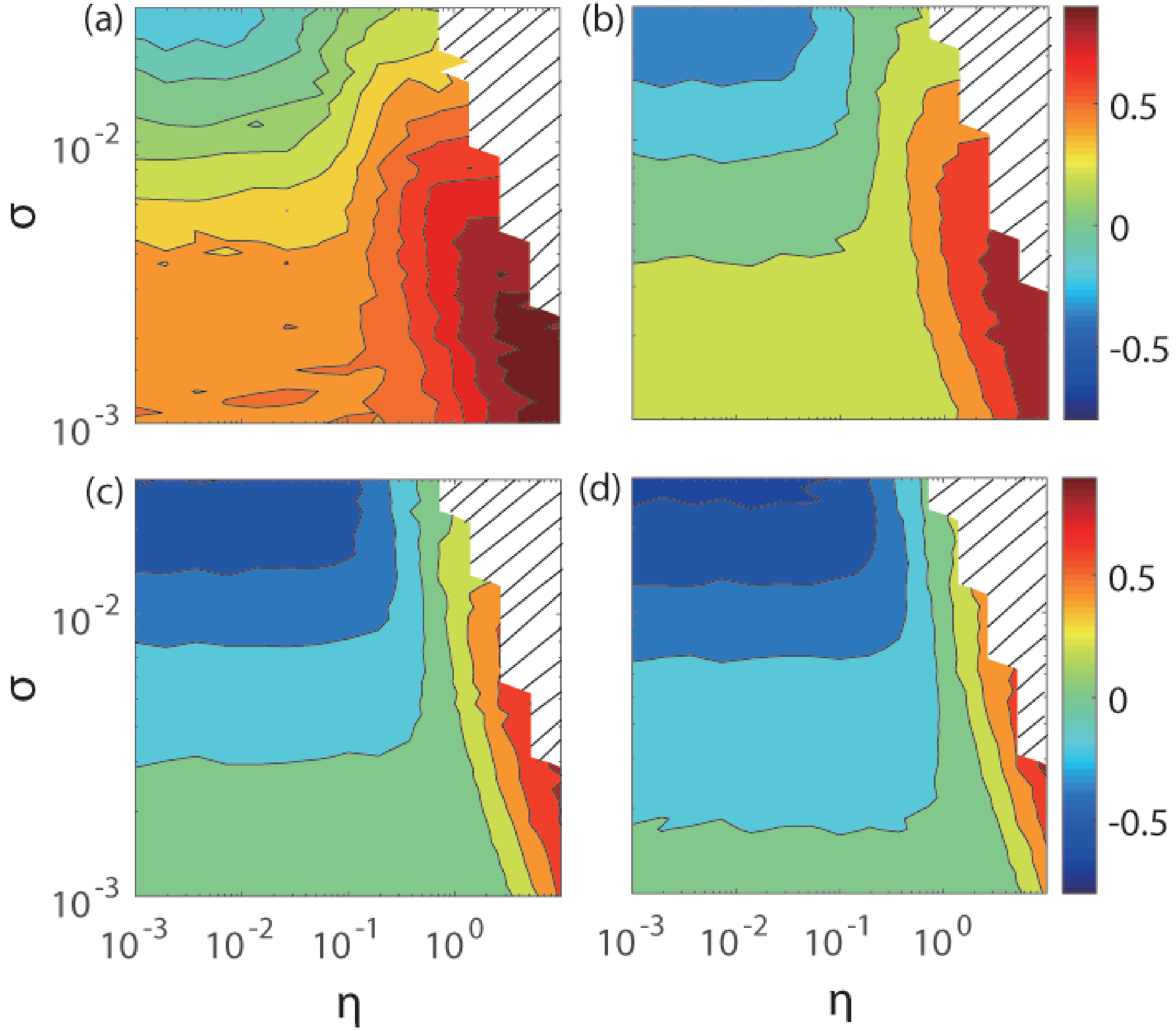

Supplementary Figure S4 | For the model predator-prey system subject to dynamical noise of amplitude σ and asymmetry ratio η, measure R in the parameter plane (σ, η). We fix $\beta_{y,x} = 0.1$ and choose a number of values of $\beta_{x,y}$: (a) 0.01, (b) 0.02, (c) 0.05, and (d) 0.07. Other parameters are $r_x = 3.8$, $r_y = 3.5$, $N = 1000$, $E_x = E_y = 2$, and $\tau = 1$. The shaded region in each panel indicates divergence of system dynamics due to large noise.

Supplementary Note 3: Effect of dynamical noise on detection of directed dynamical influence

We study the effect of dynamical noise on detection of directed dynamical influence. Consider the following two-dimensional coupled map system:

$$x(t+1) = x(t)[r_x - r_x x(t) - \beta_{x,y} y(t)] + \eta\sigma\zeta_t^x,$$
$$y(t+1) = y(t)[r_y - r_y y(t) - \beta_{y,x} x(t)] + \sigma\zeta_t^y, \tag{S1}$$

where $\beta_{x,y}, \beta_{y,x} \in [0,1]$ are the coupling parameters, $r_x, r_y \in [0,4]$, ζ_t^x and ζ_t^y are the white Gaussian noise of zero mean and unit variance, η is the asymmetry ratio characterizing the bias of noise on x relative to that on y, and σ is the noise amplitude. We calculate the value of the measure R in the noise parameter plane (η, σ), as shown in Fig. S4. We obtain essentially the same patterns as in Fig. 3, indicating that dynamical noise can also enhance the detectability of directed dynamical influence. Dynamical noise, however, is intrinsic to the system dynamics and is thus less susceptible to external adjustment/control as compared with measurement noise.

Supplementary References

1. Sugihara, G. *et al.* Detecting causality in complex ecosystems. *Science* **338**, 496–500 (2012).

2. Gause, G. F. *The struggle for existence* (Courier Dover Publications, 2003).

3. Luckinbill, L. S. Coexistence in laboratory populations of paramecium aurelia and its predator didinium nasutum. *Ecology* **54**, 1320–1327 (1973).

4. Veilleux, B. G. *The analysis of a predatory interaction between Didinium and Paramecium*. Master's thesis, University of Alberta, Edmonton (1976).

5. Jost, C. & Ellner, S. P. Testing for predator dependence in predator-prey dynamics: a non-parametric approach. *Proc. Biol. Sci* **267**, 1611–1620 (2000).
